# Laveran, le pasteurien

**DOI:** 10.48327/mtsi.v3i1.2023.313

**Published:** 2023-02-20

**Authors:** Annick Perrot

**Affiliations:** Conservatrice honoraire du musée Pasteur, 25 Rue du Dr Roux, 75015 Paris, France; * Actes du Colloque – Centenaire de la mort d'Alphonse Laveran. 24 novembre 2022, Paris / Proceedings of the Conference – Centenary of the death of Alphonse Laveran. 24 November 2022, Paris

**Keywords:** Alphonse Laveran, Paludisme, Protozoologie, Trypanosomes, Leishmanioses, Prix Nobel, Institut Pasteur, Val de Grâce, Société de pathologie exotique, France, Alphonse Laveran, Malaria, Protozoology, Trypanosomes, Leishmaniasis, Nobel Prize, Pasteur Institute, Val de Grâce Hospital, Société de pathologie exotique, France

## Abstract

Arrivé au terme de son mandat professoral au Val de Grâce, Alphonse Laveran, Médecin principal, est nommé à Lille puis à Nantes. Ces affectations le privent d'un service hospitalier pour poursuivre ses recherches et, face à la rigidité des autorités hiérarchiques, il demande sa mise à la retraite en 1896. Il a 50 ans. Le Dr Roux l'accueille à l'Institut Pasteur, en qualité de chef de service honoraire, où, bénévolement, il commence une deuxième carrière. Il va consacrer 25 ans de travaux aux grands problèmes de protozoologie. Avec Félix Mesnil, il entreprend l’étude de la trypanosomiase et celle des leishmanioses. En 1907, le prix Nobel de physiologie ou médecine lui est décerné pour « ses travaux sur le rôle des protozoaires comme agents de maladies ». Il affectera une grande partie du montant de son prix à l'aménagement et au développement de locaux consacrés à la parasitologie à l'Institut Pasteur, où convergeront, depuis toutes les colonies, les découvertes et observations faites dans ce domaine. En 1908, il fonde la Société de pathologie exotique. Il consignera ses mémorables recherches dans pas moins de 600 publications. Malgré son apparente rigidité, l'homme se révélait d'un abord aimable par « la simplicité de ses manières, son aménité et son grand cœur ».

## Abstract (en)

At the end of his professorial mandate at the Val de Grâce hospital, Alphonse Laveran, who was Chief Doctor, was appointed to Lille and then Nantes. These assignments however deprived him of a hospital service where he could carry on his research. In 1896, faced with the inflexibility of his military hierarchy, he applied for early retirement; he was then 50 years old. Dr Roux welcomed him to the Pasteur Institute as a honorary department head, where he started a second career as a volunteer. He would spend the following 25 years there, addressing the great protozoology issues. Together with Félix Mesnil, he undertook the study of trypanosomiasis and leishmaniases. In 1907 he was awarded the Nobel Prize in physiology and medicine to honor “his work on the role of protozoa as pandemic vectors”. He allocated a large part of his prize money to set up a parasitology laboratory at the Pasteur Institute, where all the discoveries and observations carried out in the colonies would converge. In 1908, he funded the Society of Exotic Pathology. He authored no less than 600 publications where his memorable research is recorded. Despite his somewhat outwardly rigid appearance, the man proved to be of amiable approach thanks to “the simplicity of his manners, his amenity and his great heart”.

En décembre 1921, lors de la traditionnelle cérémonie des vœux, le Dr Roux, Directeur de l'Institut Pasteur, disait aux pasteuriens réunis autour de lui: « C'est avec un profond regret que nous ne voyons pas auprès de nous aujourd'hui notre maître à tous, M. Laveran qui est la gloire vivante de cette maison. Il est retenu chez lui par son état de santé; quelques-uns d'entre nous iront dans un instant lui porter les vœux ardents de cette assemblée pour son rétablissement. » Hélas, Laveran décédait l'année suivante, le 18 mai 1922.

Cent ans plus tard, l'Institut Pasteur s'honore d'avoir compté Alphonse Laveran parmi les plus illustres de ses chercheurs. Mon intervention veut évoquer ce qu'on peut appeler la « seconde partie » de son parcours, œuvre toute consacrée à la parasitologie médicale.

En 1896, à 50 ans, il demandait sa mise à la retraite du Service de santé de l'Armée, achevant, avec quels succès, la « première partie » qu'il avait accomplie comme médecin traitant et homme de laboratoire aussi bien au Val de Grâce qu'en Algérie [1]. Il quittait l'armée, non sans quelques remous, essuyant des vexations des autorités hiérarchiques qui appliquaient étroitement les règlements militaires, mais aussi jaloux de sa notoriété.

En effet, arrivé au terme de son mandat professoral au Val de Grâce, en 1894 (Fig. 1), « il souhaitait, comme le rappelle (dans son article nécrologique) le Dr Louis Vaillard, médecin général inspecteur, une résidence qui lui permît, sans nuire à ses fonctions, de confirmer ses recherches – désirs inopportuns pour certains dirigeants du jour auxquels portaient ombrage le caractère et l'indépendance du savant ! ».

**Figure 1:**
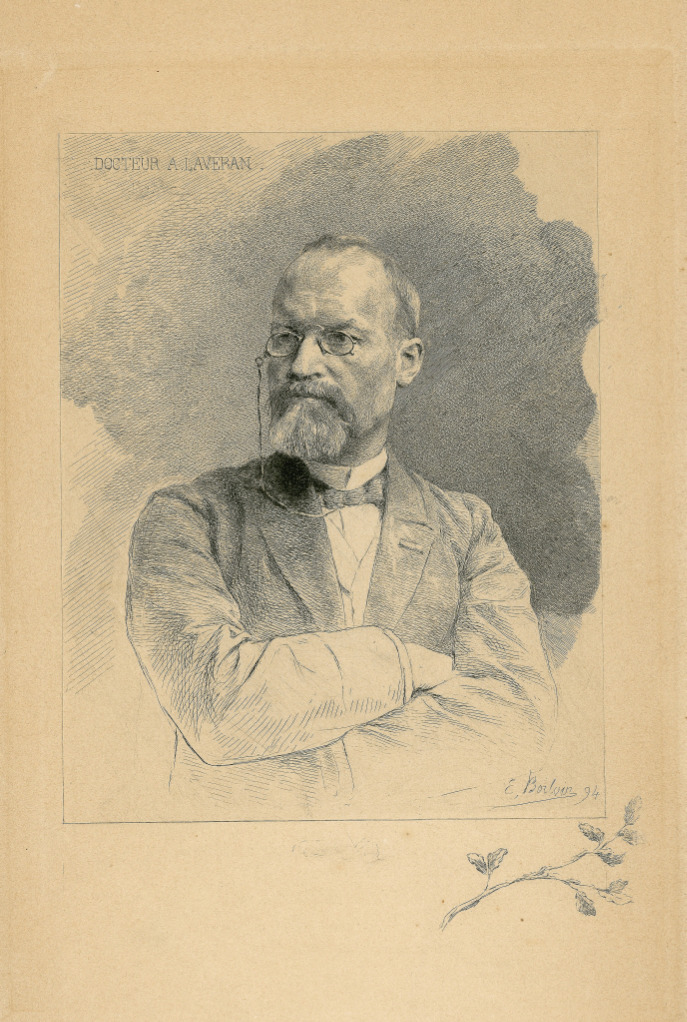
Alphonse Laveran, dessin gravé d’Émile Boilvin, 1894 (crédit photo: Institut Pasteur/Musée Pasteur) Alphonse Laveran, engraving by Émile Boilvin, 1894 (photo credit: Institut Pasteur/Musée Pasteur)

Nommé médecin en chef de l'hôpital de Lille, puis à la direction du service de santé du XI^e^ Corps d'armée à Nantes, non seulement il est privé d'un service hospitalier susceptible d'alimenter ses recherches, mais ses occupations ne lui procurent qu'un profond ennui. L'homme, au caractère affirmé, estimait qu'il perdait son temps… Ni patient ni résigné, il prend la décision d'une retraite prématurée et d'aller porter sa renommée ailleurs.

La découverte de l'hématozoaire l'avait rendu illustre. Ses nombreux travaux lui valaient une renommée quasi internationale. Pourtant il recherchait le calme et la sérénité nécessaires pour développer ses recherches. L'Institut Pasteur était bien le lieu propice à lui offrir ces conditions de travail dans une complète indépendance d'esprit.

Or ce n’était nullement une *terra incognita*; il pourrait y renforcer des liens tissés depuis longtemps. En effet, on trouve Laveran auditeur au premier cours de microbie technique en 1889 [5] et parmi la délégation pasteurienne qui accompagnait Roux au congrès d'hygiène de Budapest en 1894, où Roux donnait les résultats de la sérothérapie. Et, souvenons-nous, Roux fut élève de Laveran au Val de Grâce avant de s'en faire exclure… « pour bricolage biologique ».

À 51 ans donc, Émile Roux l'accueille cordialement à l'Institut Pasteur où, selon Émile Duclaux, « il apportait les conseils de son expérience et l’éclat de son nom ». Il est nommé chef de service honoraire.

S'il apportait à l'Institut Pasteur sa renommée, faut-il le rappeler, il se voulait bénévole – en démissionnant, il avait sacrifié ses intérêts matériels, sa retraite proportionnelle, minime, toutefois compensée par ses propres revenus et ceux de sa femme, qui semblent lui assurer son quotidien. Il se contente d'une installation modeste, hébergé dans le service d’Élie Metchnikoff, partageant une pièce avec Félix Mesnil, autre maître de la parasitologie, avec lequel il va mener une longue et féconde collaboration qui effacera les tensions des premières années de la cohabitation ! Le lieu était modeste, mais l'environnement intellectuel de haute qualité (Fig. 2).

**Figure 2:**
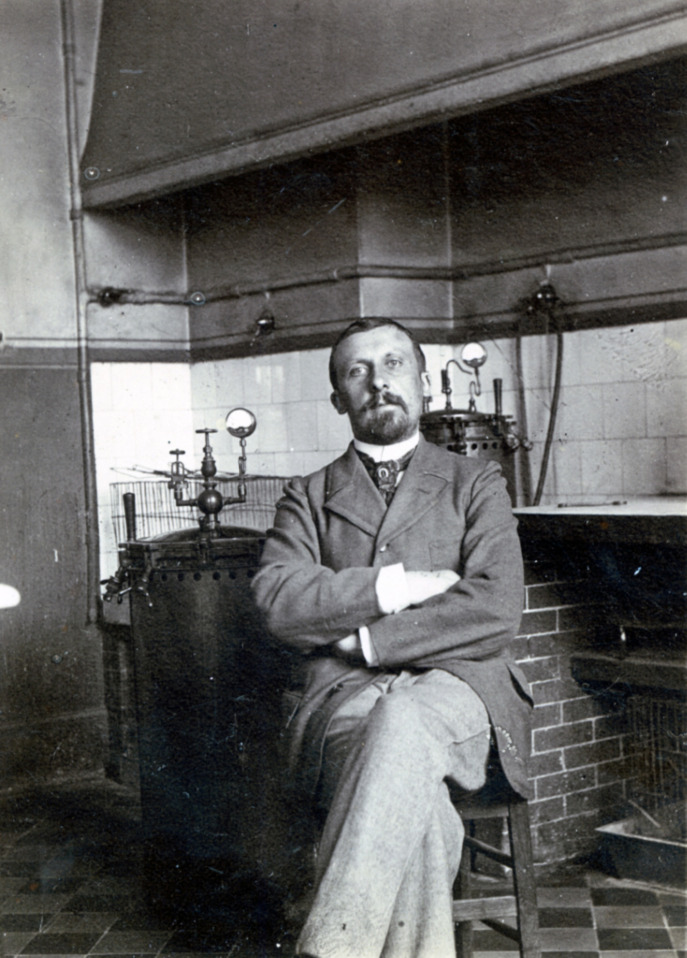
Félix Mesnil (1868-1938) dans le laboratoire d’Élie Metchnikoff, Institut Pasteur, v. 1910-1920 (crédit photo: Institut Pasteur/Musée Pasteur) Félix Mesnil (1868-1938) in Élie Metchnikoff's laboratory, Pasteur Institute, c. 1910-1920 (photo credit: Institut Pasteur/Musée Pasteur)

Ainsi, dès son arrivée en 1896, il entame une nouvelle et intense période d'activité scientifique qui se poursuivra jusqu’à sa mort. Pendant 25 années, il imprimera sa marque magistrale aux grands problèmes de protozoologie dont la solution enrichit autant la science fondamentale que la santé publique.

Sur 600 publications qui constituent son œuvre, 440 ont été faites à l'Institut Pasteur. Il reprend ses recherches sur les protozoaires en les étendant à un vaste ensemble d'animaux: oiseaux et animaux à sang froid. Il identifie plusieurs espèces nouvelles d'hématozoaires dont le nombre s'accroît au point qu'il en donne une classification.

Mais le paludisme ne disparaît pas de ses préoccupations !

Il aurait renié sa formation de médecin militaire s'il n'eût été avant tout épidémiologiste. Et son implication trouve comme un écho dans la parole de Pasteur: « J'avoue que je n'ai jamais songé, en pensant à une maladie, à lui trouver un remède mais toujours au contraire, à trouver une méthode capable de la prévenir. » Il n'ignore aucune des questions d'hygiène, et prend une part active à la prophylaxie du paludisme, notamment en Corse [4]. En 1907, il publie la 2^e^ édition enrichie de son *Traité du paludisme*, dont la 1^re^ édition date de 1898.

À partir de 1900, Laveran se consacre particulièrement aux trypanosomes, agents de diverses épizooties et maladies humaines, notamment la grave endémie africaine, la maladie du sommeil, découvrant au moins une trentaine d'espèces nouvelles.

Ces études, qu'il mène seul ou avec Félix Mesnil, confèrent un éclat particulier à l'Institut Pasteur: thérapeutique des trypanosomiases. La somme de leurs connaissances se trouve exposée en 1904 dans l'important traité *Trypanosomes et trypanosomiases*, qu'ils compléteront dans une 2^e^ édition en 1912.

En 1903, un nouveau champ d’études s'offre à Laveran: les leishmanioses. Avec Mesnil, et après Leishman et Donovan, il décrit l'agent du kala-azar, une endémie redoutable sévissant en Inde et connue aujourd'hui sous le nom de *Leishmania donovani.*

« Ces travaux (notamment sur les leishmanioses et les trypanosomiases), a dit Noël Bernard, qui sont d'un si grand intérêt pour la pathologie comparée et la pathologie générale, marquent la continuité de la pensée de Laveran, par leur enchaînement logique et rigoureux. Chacun d'eux eût suffi à assurer la notoriété scientifique de leur auteur. »

Enfin, en 1907, la grande valeur de ses travaux est reconnue et couronnée par le prix Nobel, décerné « pour ses travaux sur le rôle des protozoaires comme agents de maladies ». Alphonse Laveran est le premier Français à qui est attribué le prix Nobel de physiologie ou médecine.

Aussitôt, il veut consacrer une grande partie du montant de son prix à l'installation d'un laboratoire. Voici ce qu'il écrit au Président du Conseil d'administration de l'Institut Pasteur [8]:

« Paris, le 22 décembre 1907.

Mon Cher Confrère et Ami,

Mon vœu le plus cher était d'apporter une pierre à la Maison de Pasteur où j'ai été si bien accueilli lorsque j'ai quitté la médecine militaire. Grâce au prix Nobel de médecine qui vient de m’être décerné, ce vœu peut être exaucé. Je vous prie d'accepter pour l'Institut Pasteur une somme de cent mille francs. Je désire que cette somme soit employée à l'installation ou à l'entretien d'un petit laboratoire destiné spécialement à l’étude des protozoaires pathogènes.

Veuillez agréer, Mon Cher confrère et ami, l'assurance de mes sentiments les meilleurs et les plus dévoués.

Signé: A. Laveran »

Le Conseil d'administration venait d'acheter sur le fonds Osiris un terrain et une maison au 96, rue Falguière et de vastes écuries et remises ayant appartenu à la Compagnie des voitures de place « L'Urbaine » [2]. Sans tarder, on installe dans cet immeuble ce qui deviendra « le laboratoire Laveran » ou « le laboratoire des Maladies tropicales » (Fig. 3) [3]. Le deuxième étage est réservé à Laveran, les autres pièces sont destinées au chef de laboratoire, aux assistants, et les annexes sont dévolues aux animaux: écuries, chenil, volière, aquariums… Ce qui frappait dans le laboratoire de Laveran, se souviendra Émile Roubaud, « c’était l'ordre, la lumière et la propreté ». Il le qualifie de « centre de labeur discret et silencieux ».

**Figure 3:**
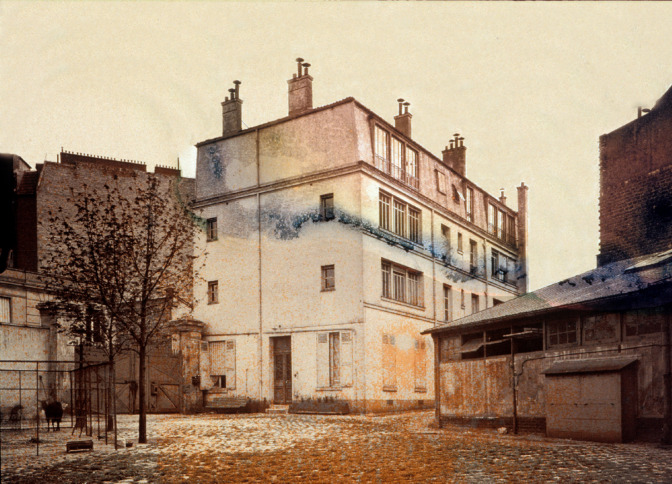
Bâtiment Alphonse Laveran à l'Institut Pasteur, v. 1910-1920. D'après un autochrome couleur (crédit photo: Institut Pasteur/Musée Pasteur) Alphonse Laveran building at the Pasteur Institute, c. 1910-1920. After a color autochrome (photo credit: Institut Pasteur/Musée Pasteur)

Dans cet empire colonial français de l’époque en plein développement, le corps du Service de santé des troupes coloniales se trouvait confronté aux pathologies locales, la plupart engendrées par des protozoaires, et aux insectes vecteurs.

Aussi le laboratoire leur ouvre-t-il largement ses portes ! Effet éminemment attractif d'un laboratoire où règne Laveran, et auprès de lui Mesnil pour les études des protozoaires, puis Marchoux pour ses études des bactéries, ensuite Roubaud dans le service d'entomologie. Sorte de creuset, lieu d’échange entre l'Institut Pasteur et les laboratoires des colonies. Les médecins accourent, viennent consulter, apprendre, échanger le fruit de leurs observations.

Travailleur plutôt solitaire, Laveran ne s'entoure que transitoirement de collaborateurs. Parmi eux le fidèle André Thiroux, qui quittera un temps Tananarive, puis David Roudsky, engagé volontaire qui sera tué au front en 1916. Et le légendaire aide-préparateur Léon Breton, formé par les soins du grand Patron auquel il vouait un inaltérable attachement.

D'un abord distant, d'une froideur apparente, la personne inspire respect voire crainte, et il s'impose une discipline inflexible. Pas étonnant que les coloniaux de passage s'attardent moins dans son laboratoire, lui préférant celui de Mesnil, plus avenant !

On lui accorde pourtant de grandes qualités de cœur, et Roubaud témoigne encore: « Le trait dominant qui demeure de lui, c'est, sous son apparente rigidité, une simplicité aimable et souriante et une délicatesse qui a frappé tous ceux qui ont pu l'approcher » [6]. Esprit médical, patient, méthodique, persévérant dans l'observation biologique et l'expérimentation, sa première démonstration du pouvoir pathogène d'un microbe protozoaire illustre parfaitement sa haute conscience scientifique, sa ténacité. Souvenons-nous combien il lui fallut d'opiniâtreté pour vaincre le scepticisme ambiant.

« La parole lente et sobre – esprit mesuré, prudent et juste » comme l’écrit Marie Phisalix [7], sa biographe et amie, Laveran fut un enseignant à la remarquable précision aux cours de l'Institut Pasteur en donnant les leçons sur le paludisme, les trypanosomes, les leishmanies. Avec tout le poids de son autorité universellement reconnue.

L’étude capitale des trypanosomes et des trypanosomiases, avec la collaboration de Félix Mesnil, les conduit à assurer l'organisation scientifique de la Mission française du Congo de 1906 à 1908 [7]. Trois de leurs élèves composent cette mission: Gustave Martin, Roubaud et Lebœuf, véritable annexe du laboratoire, vaste champ d'application de toutes les données acquises pour lutter contre la maladie du sommeil.

Il estime « qu’à aucun moment l’étude des maladies exotiques ne s'est imposée avec plus d’évidence en raison de l'extension des Empires coloniaux (nous sommes en 1908…), en raison aussi de la multiplicité et de la rapidité des moyens de transport qui favorisent la dissémination des maladies (toujours d'actualité !) »

Fort de cette conviction, il fonde en 1907 la Société de pathologie exotique, devenue depuis 2019 la Société francophone de médecine tropicale et santé internationale, qui a eu son siège à l'Institut Pasteur jusqu'en 2009, et en dirige les travaux pendant 12 ans, lui donnant un incontestable prestige.

Pendant la Grande Guerre, dès l'installation des troupes en Macédoine, il répond à l'inquiétude du Service de santé des Armées en confirmant début janvier 1916: « Le paludisme est endémique dans une grande partie de la Grèce. et l'on doit craindre que notre armée d'Orient, campée aux environs de Salonique, soit éprouvée par cette redoutable maladie. » Il rédige immédiatement des notices sur les mesures antipalustres à imposer, dont il énumère une série bien codifiée: lutte contre les larves de moustiques (antilarvaires) par assainissement des eaux stagnantes, mise en place de moustiquaires individuelles, administration préventive de quinine (quininisation) à raison de 40 cg de chlorhydrate de quinine par homme et par jour de mai à octobre. Mesures qui seront reprises par les frères Sergent sur le terrain. Laveran siègera à l'Assemblée de l'Institut Pasteur, puis au Conseil d'administration. En 1915, à l'occasion de son jubilé, on lui confèrera le titre de Directeur honoraire; il est le seul à le porter sans avoir exercé les fonctions de direction. Yersin le sera en 1934, mais lui était directeur de l'Institut Pasteur de Nha Trang.

Émile Roux prenait conseil auprès de son ancien maître. Tout le monde reconnaissait sa vaste érudition, louait sa probité d'esprit, sa droiture, sa générosité et sa modestie, mais aussi le travailleur infatigable.

Un de ses biographes a rappelé que, dans les derniers temps de sa vie, obligé de garder le lit, il s'organisait pour pouvoir encore examiner des préparations au microscope. Comme l'a dit le Professeur Vaillard, « la mort seule a pu clore la journée de ce grand ouvrier ».

J'ai fait appel aux citations de pasteuriens qui ont été proches de Laveran pour tenter d'approcher un court moment son œuvre magistrale. Je laisserai donc la conclusion à Albert Calmette qui est le plus bel hommage: « Avant Laveran, a-t-il écrit, personne n'avait soupçonné le rôle pathogène des hématozoaires, de sorte qu'il n'est pas exagéré de dire que son œuvre apparaît aujourd'hui la plus importante après celle de Pasteur. »

## Liens D'intérêts

L'auteure ne déclare aucun lien d'intérêt.
